# Roger’s diffusion of innovations theory and the adoption of a patient portal’s digital anamnesis collection tool: study protocol for the MAiBest project

**DOI:** 10.1186/s43058-024-00614-8

**Published:** 2024-07-15

**Authors:** Florian Wurster, Paola Di Gion, Nina Goldberg, Volker Hautsch, Klara Hefter, Christin Herrmann, Georg Langebartels, Holger Pfaff, Ute Karbach

**Affiliations:** 1 Chair of Quality Development and Evaluation in Rehabilitation, Institute of Medical Sociology, Health Services Research and Rehabilitation Science, Faculty of Human Sciences & Faculty of Medicine and University Hospital Cologne, University of Cologne, Cologne, Germany; 2 Department of Digital Clinical Systems, Clinical Affairs and Crisis Management Unit, University Hospital Cologne, Cologne, Germany

**Keywords:** Implementation Science, Digital Transformation, Patient Portal, Diffusion of Innovation, Hospital, Germany

## Abstract

**Background:**

German hospitals are legally obliged to implement digital patient portals within the next years. Systematic reviews show that the use of patient portals may be associated with improved patient-centeredness and workflows. However, mandatory digital healthcare innovations are sometimes not used by the target group as planned or even completely rejected. Based on Roger's theory of innovation diffusion, it can be assumed that the time factor is of particular importance for the adoption of the patient portal. The aim of the project is to assess determinants of patient portal adoption and to examine whether Roger’s theory can be confirmed.

**Methods:**

The project investigates the use of the patient portal in three different clinics of a large academic teaching hospital in Germany using a longitudinal study design with three cross-sectional time points (pre, post, post). Doctors and patients are surveyed about factors that predict the use of the patient portal and whether the strength of these factors changes over time. They are also interviewed about possible barriers they experience when using the patient portal or about the reasons why the patient portal is not used. Regression models and content analyses are used to answer the research questions.

**Discussion:**

Determinants of patient portal use will be discussed under the light of the temporal component of Roger's theory. At the same time, it is expected that some determinants will remain unchanged over time. Identifying determinants independent of time allows targeting the groups, enabling specific communication strategies to empower these groups to use the patient portal, contributing to an equal health care system.

**Trial registration:**

The study was prospectively registered in the German register of clinical trials (DRKS00033125) in May 2024.

Contributions to the literature

- Literature shows that digital innovations are sometimes not adopted by the target groups despite being implemented to a high standard.
- Roger's theory of the diffusion of innovations describes different groups of people (e.g. innovators and laggards) who are ready to adopt an innovation at different times. This study analyses the adoption of a patient portal against the background of the theory described.
- The results will contribute factors to the literature that influence the adoption of a patient portal depending on the time factor and provide empirical findings on the transferability of the theoretical models to the practical example of patient portals.

## Background

### Relevance

The German healthcare system is hardly digitized. In the last representative European benchmark, Germany was ranked 16th out of 17 compared countries (Bertelsmann [5]). This is especially noticeable for the inpatient setting. In the latest national digital maturity survey, German hospitals scored an average of just 33 out of a possible 100 points [1]. Several context-specific factors can be considered to explain this. First, the German healthcare system is characterized by self-administration, which goes hand in hand with a high level of bureaucracy [7]. Secondly, the reimbursement system is dualized, with insufficient public funds for increasing investment requirements, leading to an investment backlog, particularly in digital infrastructure [11]. Not only the Covid-19 pandemic, but also excessive increases in expenditure have highlighted the need for the healthcare systems digital transformation and have led to legal action. In a series of laws (PDSG, KHZG), politics have now mandated hospitals to implement various digital innovations, while simultaneously providing significant financial resources to cover these investments.

A past example is the obligation for statutory health insurances to offer their patients a voluntary electronic health record or to process medical prescriptions electronically between prescribers and dispensing pharmacies. A scenario still to come for German hospitals is the mandatory implementation of patient portals (PP) for a digital admission, treatment and discharge management to enable a digital exchange of information between service providers and recipients by the beginning of 2025 (KHZG §19 (2)). Due to the wide range of possible functionalities (e.g. appointment booking, record access or health diary), PP offer numerous advantages for patients and organizations [12]. Systematic reviews indicate that PP can enhance patient-centered care and improve healthcare processes [2, 21, 29]. Patient-centered care, in this context, focuses on empowering patients to take a more active role in the treatment process and view themselves as partners in their care [15, 18]. Regarding healthcare processes, there is evidence for a decrease in general information requests [16] and specific telephone contacts [17, 34]. Furthermore, it is noted that documents provided by patients through the PP can enrich the healthcare process with crucial information [3].

To achieve the potential improvements, offering a PP alone is not sufficient. The given example of the obligation for statutory health insurers to offer their patients a voluntary electronic health record illustrates this. These electronic health records must be actively requested by each individual patient in order to be set up (opt-in). In 2019, before the law came into force, 62% of the patients with statutory health insurance declared their willingness to use the electronic health record [6]. However, only 0.5% had actually requested it by the end of 2021 [28]. The aim of the project presented is to prevent the example described above, for the PPs still to be implemented, that a politically enforced digital innovation is not utilized. It is therefore important to understand which factors influence the adoption of the PP and which barriers are experienced, in order to actively empower and support disadvantaged groups of people to use the PP.

The framing use case for the research project is the PPs tool of digital anamnesis collection (DAC). DAC refers to the approach of postponing the medical history taking before the actual appointment. With the help of the PP, the patients can use their computer or smartphone to enter their medical history, allergies, etc. at home at their own pace or with their relatives’ support. This is meant to improve the quality of the information, which is also available for the physician when planning the appointment and therefore allows the consultation to be more efficient and target-oriented [27]. On the other hand, the DAC is characterized by the fact that it must be equally used by both, patients but also the physicians in order to realize its potential to improve healthcare delivery, making it even more important to understand the described factors, influencing the adoption of the PP. Of particular relevance is the fact that all German hospitals will be required to offer such DAC in the future.

### Theory

**Fig. 1:**
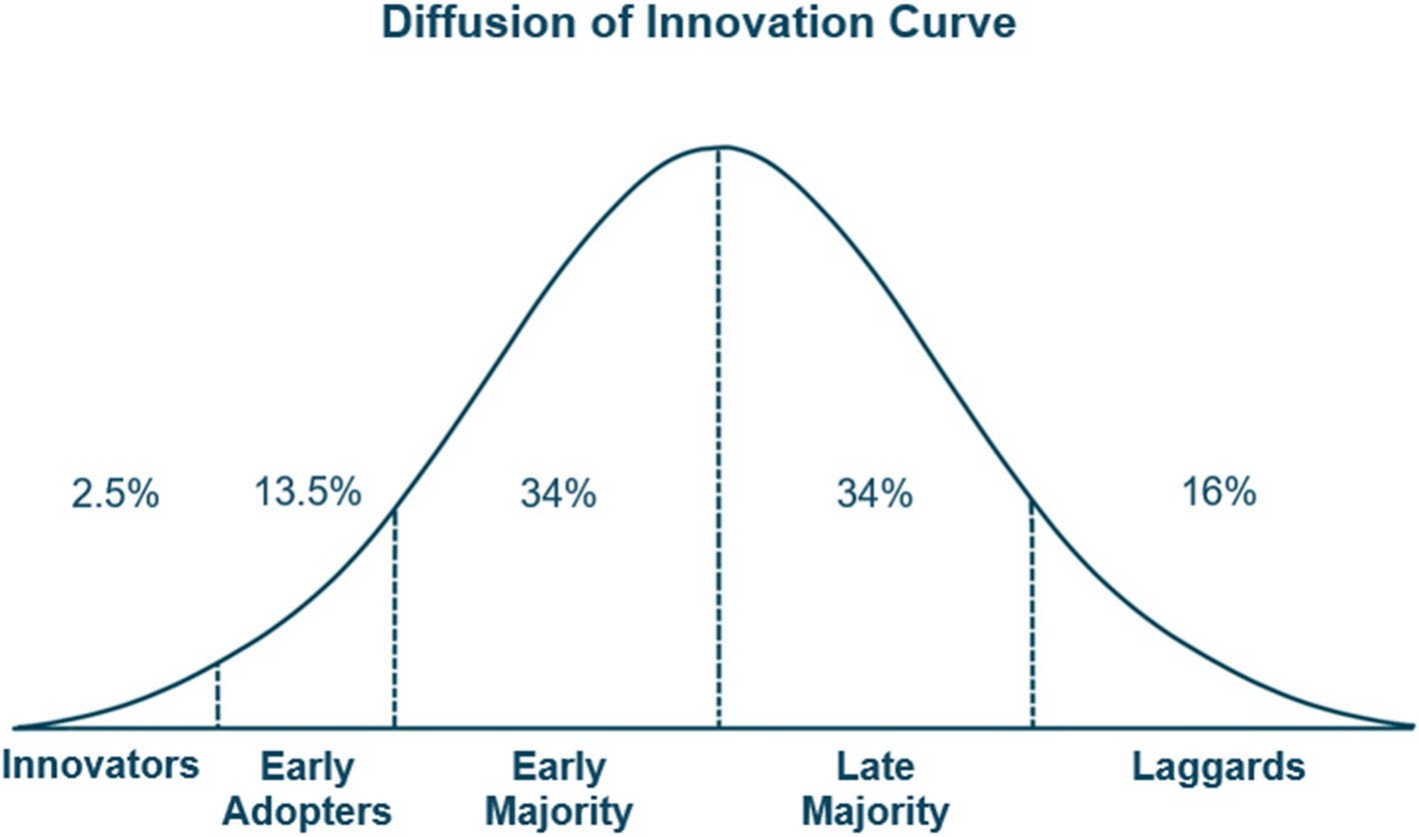
Temporal diffusion of innovations [25]

Roger's theory of the diffusion of innovations (DOI) serves as a framework for investigating the determinants of the adoption of the PP [25]. The DOI describes various factors (person-related or innovation-related) that influence when and whether an individual is willing to use an innovation. In that matter, the dimension of time is of particular relevance. In this temporal sense, the DOI distinguishes five types of "innovation adopters" (*innovators, early adopters, early majority, late majority, laggards*) who adopt an innovation at different points in time (Fig. 1). The *innovator* type, a very small group, is interested in using innovations as early as possible, while the *laggard* is unwilling to use the innovation until a long time has passed, if the innovation can still be called an innovation for the *laggards*, in view of the time that has passed since its introduction until the *laggard* type is ready to adopt.

Assuming, for example, that the individual’s digital literacy is a possible determinant of PP usage, it is expected that individuals with a markedly high level of digital literacy will adopt the PP particularly early (thus corresponding to the *innovators* type). However, the relevance of high digital literacy as a determinant of PP usage decreases over time. Accordingly, at a later stage, individuals with lower digital literacy would also adopt the PP (e.g. representing the *late majority* type). Regarding the research topic of PP, it is unclear whether the DOI can be applied. Even though one study has already shown that, following the DOI, the proportion of users of the PP increased slowly but steadily over time, implying an expected transition from only *innovators* among the users to additional *early adopters* among the users at a later date [33]. However, it is still unexplored, whether the relevance of potential determinants weakens over time or remain time constant. At the same time, it is uncertain whether and how the strength of the determinants or the extent of the expected weakening of these determinants is related to the clinical context (e.g., outpatient/inpatient) in which the PP is offered for use. In addition, there is no conclusive evidence as to whether and how the implementation of a PP and particularly the DAC affects documentation and treatment quality.

### Hypotheses & research questions

All research questions explicitly addressed in the research project are listed in Table 1.

**Table 1:** Dedicated hypotheses & research questions

Quantitative Hypotheses (A) Determinants of Patients PP Usage	
A1	The relative proportion of patients using DAC with low digital literacy increases over the observation period
A2	The relative proportion of patients using DAC with low health literacy increases over the observation period
A3	The relative proportion of patients using DAC with low technology acceptance increases over the observation period
A4	Patients planned for inpatient treatment are more likely to use DAC than patients receiving acute outpatient care
A5	Patients who are frequently treated in the hospital (either outpatient or inpatient) are more likely to use DAC than those who are less frequently treated
Quantitative Hypotheses (B) Determinants of Physicians PP Usage	
B1	The relative proportion of physicians using DAC with low digital literacy increases over the observation period
B2	The relative proportion of physicians using DAC with low technology acceptance increases over the observation period
Qualitative Research Question (C) Barriers to PP Usage or Reasons for Non-Usage	
C1	What are the patient-related reasons for non-usage and barriers during the usage of DAC over time?
C2	What are the physician-related reasons for non-usage and barriers during the usage of DAC over time?
Exploratory Research Question (D) Actual PP Usage	
D1	How does the documentation of medical history change over time with the utilization of DAC through the PP?
Exploratory Research Question (E) Changes in Administrative Data	
E1	What changes occur in the data collected for administrative purposes after the introduction of the PP?

## Methods / design

**Fig. 2:**
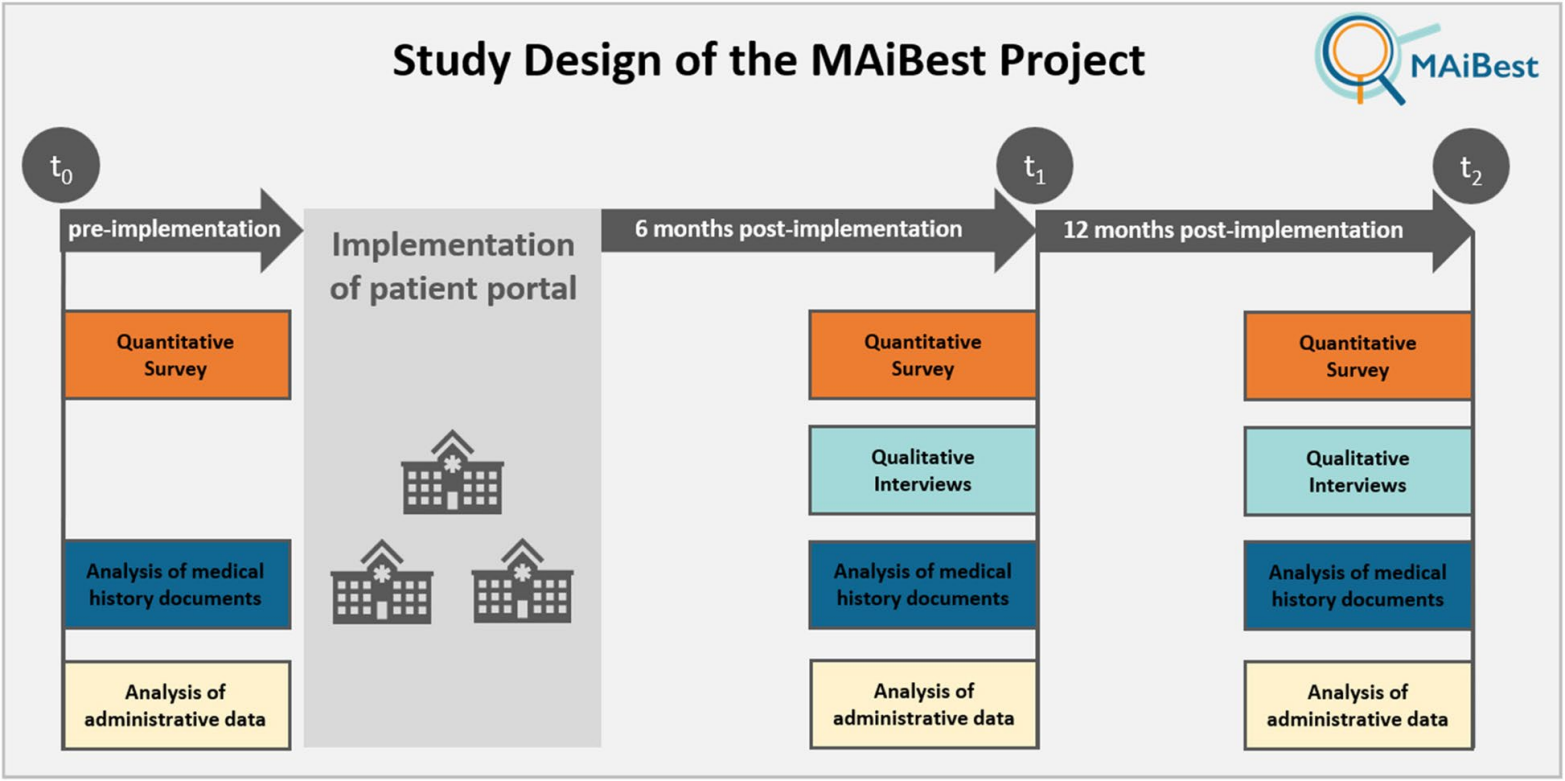
MAiBest study design

The study follows a longitudinal triple cross-sectional design (pre-post-post). It is based on three consecutive data collection phases, conducted in three clinics of a large academic teaching hospital in Germany. Data will be collected in each of the three clinics 2 months before the implementation (t0), as well as 6 months (t1) and 12 months (t2) after the implementation of the PP. Each of the three clinics has an eight-week period allocated for data collection to achieve the targeted case numbers described below. Quantitative data from surveys of patients (A) and physicians (B), qualitative data from interviews with patients and physicians (C), as well as medical history documentation (D) and administrative data from a participating clinic (E) are used to answer the research questions (Fig. 2). Each of the research questions (A) – (E) will be presented in detail in the following, including detailed case number calculations or sampling strategies.

A) Analysis of determinants of patients PP usage

The trend survey at the three measurement time points (t0; t1; t2) allows for the depiction of determinants of PP usage and the expected changes in these determinants over time. The t0 survey captures the clinic-specific heterogeneity of patient-related determinants for PP use and enabling an adjustment of the trend analysis for these baseline values from t0. This facilitates the examination of trends within each clinic and allows for comparisons between the three clinics. Patients treated in the three clinics at the three measurement time points (t0; t1; t2), whether inpatient or outpatient, will receive a paper-based questionnaire with additional, optional access to a digital version. These questionnaires include items measuring patient-related determinants and, additionally, usage behavior in t1 and t2. The patient surveys are based on existing and validated scales (e.g. digital literacy [26], technology acceptance [20], or if not possible, on items developed according to methodological standards [23]. Additionally, socioeconomic and sociodemographic factors (e.g., age, gender, education, income) are recorded for later adjustment.

The sample size calculation results in a required rounded *N* = 200 patients per measurement time point per clinic (pooled total *N* = 1800). This is based on an assumed weak effect size for Cohen's D of 0.2 with 80% power and a significance level of 0.05 for a (1) two-sided independent two-sample t-test for the comparison between clinics at each measurement time point (total *N* = 393 at each time point for the comparison between two clinics, rounded *n* = 200 per clinic) and (2) for the comparison within the clinic across measurement time points in a two-sided paired t-test (required *n* = 198 per time point). The data analysis is performed using descriptive and inferential statistics, adjusting for previously collected socioeconomic and sociodemographic characteristics, conducted with the statistical software SPSS^©^.

B) Analysis of determinants of physicians PP usage

All physicians working in the three clinics at the three measurement time points (t0; t1; t2) receive digital questionnaires. These questionnaires include questions to measure physician-related determinants and additionally, in t1 and t2, questions regarding usage behavior. The surveys of physicians are based on already existing and validated scales (e.g. digital literacy [26], technology acceptance [20], or, if not possible, on items developed according to methodological standards [23]. Additionally, socioeconomic and sociodemographic factors (e.g., age, gender, work experience) are recorded for later adjustment. Due to the legal situation, physicians must inevitably use the PP in their everyday work. Nevertheless, they can use the PP, in this case the DAC, differently depending on which type of innovation adopter they belong to according to the DOI. For example, the *late majority* may only use the absolute basics, mandatory functionalities of the PP while the *innovators* also use additional advanced features, which may be optional to use. Therefore, in this project, the perceived benefit of the DAC is examined as the dependent variable, instead of the binary actual use, as it is done for the patients. Due to the organizational pressure to use, a stronger effect on physician PP usage can be expected. Based on an assumed effect size of Cohen’s D = 0.35, a power of 80%, and a significance level of 0.05 in a two-sided paired t-test, the required sample size is *N* = 66 physicians per time point, cumulated across the three clinics. A comparison of physicians between clinics at each time point will not be conducted due to the otherwise higher required sample size. The invitation to participate and access to the digital questionnaire will be distributed through internal channels, emphasizing the practical relevance of the survey for the medical staff and positively influencing the willingness to participate. The data analysis will be conducted using descriptive and inferential statistics, adjusting for socioeconomic and sociodemographic characteristics, and performed with the statistical software SPSS^©^.

C) Analysis of barriers to PP usage and reasons for non-usage

Interviews will be conducted with patients who are treated on an outpatient or inpatient basis at the three clinics during the measurement time points t1 and t2 and used or did not use the digital anamnesis. In addition, interviews will be conducted with physicians who are working on an outpatient or inpatient basis at the three clinics during the measurement points t1 and t2 and used or did not use the digital anamnesis. Based on Rogers' DOI [25], the reasons for non-usage and experienced barriers during the use of the DAC can change with the PPs increasing existence. Therefore, interviews will be conducted at both t1 and t2. The semi-structured interviews will include questions regarding the subjectively perceived barriers during the usage or reasons for non-usage.

In each of the three clinics, a total *n* = 12 interviews with patients will be conducted at each measurement time point (t1 & t2). Purposeful sampling will be employed, varying different patient characteristics (e.g., age, education) to achieve theoretical saturation [22]. The total of *N* = 72 interviews will be conducted either in person, for example, during treatment at the clinic's premises, or by telephone according to the participants' preference. Moreover, *n* = 4 interviews with physicians will be conducted in each of the three clinics at each measurement time point (t1 & t2). Purposeful sampling will be employed, varying different physician characteristics (e.g., age, specialty, ambulatory/inpatient setting) to achieve theoretical saturation [22]. The total of *N* = 24 interviews will take place in the clinics and, if possible, during working hours. This reduces the effort for the interviewees and increases the willingness to participate. Depending on the participants' preference, the interviews can also be conducted by phone. All interviews, estimated to last 45 min, will be recorded, transcribed, and anonymized. Subsequently, the data will be analyzed using content analysis [19] with the assistance of the MAXQDA^©^ 2024 software. 

D) Analysis of actual PP usage

The anamnesis documents in each of the three clinics will be analyzed at the three measurement time points (t0; t1; t2). In t0, only the physician-filled medical history will be considered, while in t1 and t2, the now via the PPs DAC tool patient-filled medical history will be examined. In each clinic and at each time point, an intentional sampling approach [22] will analyze approximately *n* = 30 documents of patients treated in the respective clinics during the survey periods. This approach results in a total of *N* = 270 documents to be analyzed. These will be examined using document analysis according to Prior [24], allowing statements about changes in the quality and content of the anamnesis documentation [4]. Haas [13] describes three functional levels of clinical documentation. In its primary function, documentation supports the preparation and monitoring of the actual treatment process. In its secondary function, documentation can also serve as a basis for billing purposes or legal obligations. In its tertiary function, documentation is used to answer scientific questions in the field of biomedicine or health services research [13]. Depending on which of the three functional levels is considered, different criteria are suitable for making statements about the quality of the documentation. For documentation in patient records, the U.S. Institute of Medicine identified completeness, readability, accuracy, and informativeness as the most important criteria for high-quality documentation [9]. Weiskopf & Weng [30] also highlighted completeness as the most important criterion for the quality of documentation in electronic patient records, especially regarding the described secondary and tertiary functions of documentation. Wurster et al. [32] further confirmed completeness as the most commonly used criterion for operationalizing documentation quality when investigating the transition from paper-based to electronic documentation and as actually receptive to change through the implementation of digital innovations [31]. In a first step, the contents of the documentation are recorded. This involves examining whether there is a change regarding the intended information to be documented (e.g., food intolerances, vaccination status) and how this information can be documented (e.g., checkboxes, free text). Based on this, an exploratory approach will be used to determine whether and how the quality of those documents can be reflected through the completeness of relevant information (as dimension of data quality). Completeness is operationalized as a percentage indicating, for each time period and clinic, whether specific information is filled in (documented vs. not documented) across all records [32]. The analysis in the pre-post-post comparison allows statements about how physician-filled and patient-filled medical histories differ and whether the expected changes over time, according to Rogers [25], are evident.

E) Analysis of changes in internal administrative and clinical parameters

In light of the fact that it is unclear to what extent the DOI can be applied to PP, it is unclear to what extent the hospital data collected for administrative purposes is suitable for making statements regarding potential improvements in care. To identify outcomes relevant for future research projects, an exploratory analytical approach is applied to the hospital data collected for administrative purposes from one participating clinic. If the utilization of PP leads to improvements in clinical outcomes, these improvements would be reflected in the administrative and clinical management data. For instance, parameters mentioned by Dumitrascu [10] such as the 30-day readmission rate, inpatient mortality, or 30-day mortality are analyzed. The data consist of administrative data for all outpatient and inpatient patients within a month at the three measurement points (t0; t1; t2). Data analysis involves descriptive and inferential statistics to compare the means of the examined parameters regarding potential differences at the three measurement points. The results provide initial indications of whether the parameters investigated here are suitable for assessing potential impacts of the PP on clinical outcomes in further research projects.

## Discussion

The reasons for potential usage barriers are diverse and can be caused both by a suboptimal implementation process and by users themselves [8]. For the present research project, it is assumed that implementation-related factors can be largely neglected. This assumption can be made because the participating hospital possesses an institutionally anchored and experienced implementation team (Department of Digital Clinical Systems) for implementing digital innovations. In recent years, various digital innovations have been successfully implemented, including the requirements for the national telematics infrastructure and the electronic health record, the introduction of a digital fever chart, and the implementation of a patient data management system in the intensive care unit. For the planned implementation of the PP, both, patient-centered and provider-centered implementation and communication strategies were developed, for which the hospital was awarded with the "German CHANGE Award 2022" [14]. Against this background, the assumption that existing barriers do not arise from the implementation process, but are actually to be found in the respective users, can be regarded as legitimate. The results can therefore be assumed relevant for all German hospitals, regardless of their implementation strategies. Another point to emphasize with regard to the relevance of the results is the projects participatory approach. The whole project, from the research questions to the use case of DAC and the study design, has been collaboratively developed with the clinical team of the Department of Digital Clinical Systems. Through this close collaboration between science and practice, the expected results have particular utility for other hospitals during the implementation of PPs.

However, the academic teaching hospital underlying the research project might distinguish itself from other hospitals by a particularly pronounced innovation climate. It is possible that this innovation climate may be less pronounced in smaller hospitals, potentially limiting the translatability of the results. Nevertheless, the guiding theory of DOI is based on a universal claim. If the theory-driven hypotheses can be accepted and research questions are answered, a possible generalizability of the results may be derived, suggesting that those determinants and barriers proving to be time-constant could be identified across different hospitals, irrespective of their innovation climate.

## Conclusion

The anticipated results will reveal (A) patient-related and (B) physician-related determinants, influencing PP usage. Additionally, (C) barriers to PP usage and reasons for non-usage will be identified. In exploratory approaches (D) the actual PP usage on the level of documentation and (E) potential changes in administratively collected data will be examined. Given that hospitals nationwide are mandated to implement PPs and the questions pursued in this study remain unanswered at the national level, the practical significance of the findings is substantial. The results, along with derived "Dos and Don'ts" for practical implementation, will support other hospitals when implementing their PPs. Furthermore, the insights can serve as guidance for software developers in aligning the development of PPs with user requirements.

## Data Availability

Not applicable.

## Abbreviations

PP: Patient Portal
DAC: Digital Anamnesis Collection
DOI: Diffusion of Innovation
